# Association of Japanese Breakfast Intake with Macro- and Micronutrients and Morning Chronotype

**DOI:** 10.3390/nu14173496

**Published:** 2022-08-25

**Authors:** Farnaz Roshanmehr, Yu Tahara, Saneyuki Makino, Ayako Tada, Nanako Abe, Mikiko Michie, Shigenobu Shibata

**Affiliations:** 1 Laboratory of Physiology and Pharmacology, School of Advanced Science and Engineering, Waseda University, Shinjuku-ku, Tokyo 162-8480, Japan; 2 Institute of Nutrition Sciences, Kagawa Nutrition University, Saitama 350-0288, Japan; 3 Asken Inc., Shinjuku-ku, Tokyo 163-1408, Japan

**Keywords:** breakfast, Japanese breakfast, western breakfast, sleep phase, circadian clock, protein source

## Abstract

(1) Background: Dietary intake may have a remarkable effect on sleep because skipping breakfast and having a late dinner affects many sleep parameters. Breakfast is the most important meal of the day for children and adults to maintain morning chronotype. We examine whether breakfast style is associated with nutrient intake and sleep factors. (2) Methods: This cross-sectional analysis, with a large sample size of 2671 (766 men and 1805 women aged 20–60 years after data brush-up), was based on data obtained from an online survey. Correlation analysis was performed using Spearman’s rank correlation test. The Kruskal–Wallis’s test followed by post hoc Dunn’s multiple comparison test was used to evaluate the interaction between sleep factors and breakfast categories. Multiple regression analyses were performed to identify variables associated with multiple confounding factors. Dietary data were analyzed using approximately one-month average dietary records from the application. The basic characteristics of the participants (age, sex, and BMI) and other lifestyle-related factors (sleep and physical activity) were obtained accordingly. Sleep parameters including the timing of weekday sleep onset, weekday wake-up, weekend (free day) sleep onset, weekend wake-up, sleep, and midpoints of sleep phase were calculated for each participant. We categorized participants’ breakfast types into five groups: (1) Japanese meal, where breakfast may contain Japanese ingredients such as rice; (2) Western meal, where breakfast may contain bread; (3) alternating eating patterns of Japanese and Western meals; (4) cereals and supplements, where breakfast may contain cereals or supplements and energy bars; and (5) skipped breakfast (no breakfast). (3) Results: The midpoint values of the sleep phase on weekends adjusted for sleep debt on work days (MSFsc) related to chronotype were higher in women, suggesting that they may prefer eveningness. Participants with obesity, young age, and low physical activity preferred eveningness with longer sleep durations. Intake of Japanese-style breakfast was significantly associated with early wake-up time on both weekdays and weekends. Cereal-style breakfast intake was significantly associated with late wake-up on both weekdays and weekends. Intake of macronutrients such as protein, fat, carbohydrate, and sodium at breakfast time was positively and strongly associated with the intake of Japanese breakfast, whereas macronutrients were negatively associated with the intake of cereal breakfast. Among micronutrients, vitamin K was positively correlated with Japanese breakfast and negatively correlated with cereal breakfast; (4) Conclusions: Japanese-style breakfast is associated not only with morning preference but also with high intake of macro- and micronutrients.

## 1. Introduction

Dietary intake may have a remarkable effect on sleep because both diet and sleep play a crucial role in maintaining long-term health and well-being [1]. Breakfast is the most important meal of the day [2], especially for children and adults. Several studies have illustrated the health benefits associated with consuming breakfast [3,4,5]. Hence, breakfast intake may be positively associated with dietary quality, body composition, and markers of risk of chronic diseases [6]. In contrast, skipping breakfast is linked to a low-quality diet, low cognitive performance, and negative health outcomes [7,8,9]. A review of 141 articles showed that skipping breakfast is more common among men than women [9,10]. A chronotype describes the timing of the sleep–wake cycle, which varies between individuals and represents the circadian rhythm phase [11]. Studies have shown that the composition of food influences many different circadian rhythms in rodents, ranging from gene expression profiles to behavioral rhythms [12]. In this regard, studies on individuals who skip breakfast have reported that the timing of food intake is a determining factor for weight gain [13]. In Japan, dietary patterns for breakfast are categorized into two categories: rice-based, Japanese-style meals and bread-based, Western-style meals [14]. The quality of the meal is assessed using the Healthy Eating Index 2015 (HEI-2015) and Nutrient-Rich Food Index 9.3 (NRF9.3), and the results demonstrate that, for breakfast, the HEI-2015 and NRF9.3 total scores are higher in the rice-based pattern than in the bread-based pattern [15]. This study suggests that rice-based meals may be helpful for health promotion because the total score is high in rice-based meals. Ogata et al. demonstrated the relationship between skipping breakfast and circadian rhythm; when the first meal of the day was delayed by 5.5 h in the breakfast-skipping condition, the phase of a biological clock marker was delayed by 1 h [9]. In animal experiments, the timing of food intake and composition of meals are two key factors for the entrainment of the circadian system [14]. Both carbohydrate- and protein-rich foods are good nutrient sources for the entrainment of signals in peripheral clocks [16]. These studies strongly suggest that rice-based Japanese-style breakfast may be associated with circadian clock factors, owing to being a healthy meal.

To the best of our knowledge, no study has shown a comparison or the association of Japanese-style and western-style breakfast macronutrient contents with sleep parameters and the circadian clock. Therefore, we conducted a cross-sectional study to determine the association of breakfast intake and macronutrient content with sleep parameters and the circadian clock expressed by the midpoint time of sleep.

## 2. Materials and Methods

### 2.1. Subjects

This cross-sectional analysis, with a large sample size (N = 4209), was based on data obtained from an online survey [17]. The data were extracted through the phone food-log Japanese application, “Asken,” in November 2020. “Asken” is one of the top 3 popular health apps in Japan and is a food-log and food-coaching app, downloaded approximately over 6,000,000 times (https://www.asken.inc/eng, accessed on 5 November 2020) [18]. Since most users (almost 95%) use this app to reduce their body weight, and females may be more interested in their body shape than males, 70% of users of this app are female. The app provides feedback on dietary content based on the Dietary Intake Standards for Japanese as determined by the Ministry of Health, Labor, and Welfare. In the current study, an online survey was conducted via the app in addition to the available dietary records. Researchers have determined that self-reported food logs compiled by the app are reliable for research purposes [19]. The median correlation coefficient between daily paper-based nutrient intake data and data from Asken in a recent study of adult women and men was approximately 0.8 [20]. In the present study, dietary data were compared with those calculated from the National Nutrition Survey of Japan (NNSJ) [21]. Based on these findings, the dietary information used in this study was found to be reliable. Nevertheless, according to these data, the amount of protein and fat was slightly higher, whereas the amount of carbohydrates and fat was lower than that of the NNSJ. Men consumed 85.5 ± 17.2 g (Mean ± SD) of protein on a daily basis and women consumed 69.3 ± 14.3 g, which exceeded the amount of protein recommended for Japanese people (80 g for men and 60 g for women) by the Ministry of Health, Labor, and Welfare in Japan [21]. As one might expect, the findings of this study might be explained by Asken’s nature as a weight management app, which is usually intended to maintain or lose weight, and Asken’s users are generally the health-conscious and self-confident type.

The Ethics Review Committee on Research with Human Subjects at Waseda University approved this experiment (No. 2020-046), and the guidelines of the Declaration of Helsinki were followed accordingly. Informed consent was obtained from all the participants. The target population consisted of apparently healthy Japanese individuals of both sexes aged 10–70 years. We excluded all participants aged 10 and 70 years owing to limited data. In total, 1230 men and 2661 women aged 20–60 years had dietary data for inclusion in the analysis. 

### 2.2. Study Protocol

#### 2.2.1. Questionnaire

In this study, we designed a questionnaire based on participants’ information on the Asken application. The basic characteristics of the participants (age, sex, and BMI) and other lifestyle-related factors (sleep and physical activity) were obtained from an online survey. 

#### 2.2.2. Sleep Parameters and Assessment of Morning Type or Evening Type 

Sleep parameters including the timing of weekday sleep onset, weekday wake-up, weekend (free day) sleep onset, weekend wake-up, and the midpoints of sleep phase were calculated accordingly for each participant. As previously reported, our data included a normal distribution of chronotype (individual morning and evening preferences), which was calculated from the midpoint of the sleep phase on weekends adjusted for sleep debt on work days (MSFsc) and age-dependent phase-advancement of MSFsc [22]. The midpoint of sleep was calculated using self-reported bedtimes and wake times on weekdays and weekends. The midpoint of sleep on weekends was associated with circadian clock regulation, such as morning and evening preferences [23].

#### 2.2.3. Physical Activity

Physical activity was determined by the number of days and hours spent on three types of activities from an online survey (vigorous-intensity activity, moderate-intensity activity, and walking). We calculated weekly metabolic equivalents (MET) based on the International Physical Activity Questionnaire (IPAQ) analysis guidelines for the intensity of each activity, as well as the intensity of total physical activity (Committee, 2005) (https://sites.google.com/site/theipaq/scoring-protocol, accessed on 5 November 2020). 

#### 2.2.4. Dietary Data

Dietary data were analyzed using approximately one-month average dietary records from the application. The energy content (kcal), protein, fat, carbohydrate, sodium, potassium, calcium, magnesium, phosphorus, iron, zinc, vitamin A, vitamin C, vitamin D, vitamin E, vitamin K, vitamin B1, vitamin B2, vitamin B6, vitamin B12, niacin, folate pantothenic acid, and dietary fiber were measured for each of the three meals. The intake timing of snacks might have been different among participants because the snack time was not checked in this study. In our previous study, the validity of the dietary record of this app was high [24].

#### 2.2.5. Breakfast Style

We categorized participants’ breakfast types into five groups: (1) Japanese meal, where breakfast may contain Japanese ingredients such as rice; (2) Western meal, where breakfast may contain bread; (3) Japanese-Western style meal, where breakfast may contain alternating eating patterns of Japanese and Western meals; (4) cereals and supplements, where breakfast may contain cereals or supplements and energy bars; and (5) skipped breakfast (no breakfast). For each participant, the selected category of breakfast style was designated as score 1, and the other categories were designated as score 0. Therefore, breakfast-style was analyzed using a score of 1 or 0. We then calculated the amount of macronutrient and micronutrient intake during breakfast, lunch, and dinner for each participant.

#### 2.2.6. Eating Period

The eating period was scored by the time consumed to eat breakfast using a questionnaire. Score 1, very quick to consume; Score 2, quick to consume; Score 3, slow to consume; Score 4, very slow to consume.

### 2.3. Statistical Analysis

All data analyses were performed using the IBM SPSS Statistics (version 25) predictive analytics software. All data are presented as the mean ± standard error of the mean or the mean ± standard deviation of the mean. Since the normality test was not passed, the Kruskal–Wallis’s test followed by post hoc Dunn’s multiple comparison test was used to evaluate the interaction between sleep factors, breakfast categories, and protein source. Two-group differences were statistically analyzed using the Mann–Whitney test. Fisher’s exact probability test was applied to determine breakfast type choice between men and women. Correlation analysis was performed using Spearman’s rank correlation test. Multiple regression analyses were performed to identify variables associated with multiple confounding factors. The level of significance was set to 5%, and *p*-values of less than 0.05 were considered statistically significant. 

## 3. Results

This cross-sectional analysis, with a large sample size (N = 4209), was based on the data obtained from an online survey. The present analysis included 2571 Japanese adults (766 men and 1805 women) aged 20–60 years (Table 1).

The percentage of Japanese-style breakfast (BF) was higher in men and that of Japanese–Western-style BF was higher in women (Table 1). Except for weekday wake-up, the other parameters were higher in women than in men. The MSFsc values were higher in women, suggesting that women may prefer eveningness.

All data are expressed as mean ± SD, except for the Breakfast style, which is expressed as a percentage. Statistical significance was determined by Mann–Whitney analysis, except for the Breakfast style, which was analyzed by Fisher’s exact probability test. NS; *p* > 0.05. BMI, body mass index. METs and exercise parameters. J-W, Japanese and Western styles. 

To find overall associations between sleep parameters and sex, age, BMI, METs, and breakfast meal styles, we performed correlation analysis using Spearman’s rank correlation test (Table 2). Weekday/weekend sleep onset was positively associated with age and METs. Weekday/weekend wake-up and sleep duration were positively associated with sex and BMI and negatively associated with age and METs. These data suggest that participants who are female, obese, young, and have low physical activity prefer eveningness with longer sleep durations. Breakfast meal styles were associated with sleep parameters and MSFsc (Table 2). Intake of Japanese-style breakfast was significantly associated with early wake-up time on both weekdays and weekends. Cereal-style breakfast intake was significantly associated with late wake-up on both weekdays and weekends. Participants with Japanese-style BF may prefer morningness, whereas those with cereal-style BF may prefer eveningness.

**Figure 1:**
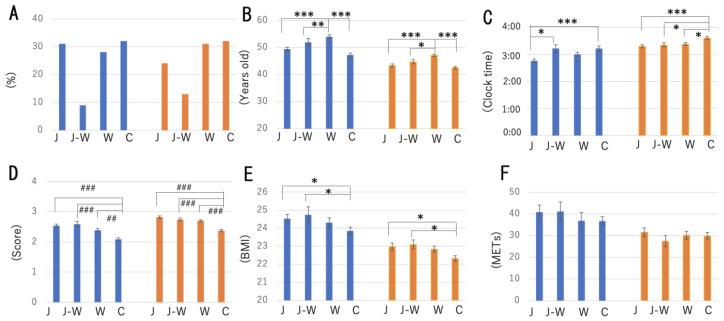
Interaction between various parameters and breakfast meal category. (**A**) Preferences for each meal category. Associations between age (**B**), MSFsc (chronotype) (**C**), eating period (**D**), BMI (**E**), exercise (total METs) (**F**), and meal category. Blue column—male; orange columns—female. Statistical significance was revealed using ANOVA followed by Tukey’s multiple comparison post hoc test: * *p* < 0.05, ** *p* < 0.01, *** *p* < 0.001, or Kruskal–Wallis’s test followed by Dunn’s multiple comparison post hoc test: ## *p* < 0.01, ### *p* < 0.001. J—Japanese meal; J-W—Japanese and Western meals; W—Western meal; C—cereal meal.

The interaction between the various parameters and breakfast meal categories is shown in Figure 1. The preference for each meal category indicates that male Japanese adults tend to consume Japanese, cereals, Western, and Japanese–Western (alternative eating patterns of Japanese and Western meals) breakfast styles. Female adults tend to consume cereals and Western, Japanese, and Japanese–Western breakfast styles (Figure 1A). Additionally, the correlation between the consumption of different types of breakfast and age was examined (Figure 1B). Female participants were approximately six years younger than male participants (Figure 1B, Supplemental Table S1). In both sexes, participants who preferred Western-style breakfast were of older ages, whereas cereals were preferred by younger participants (Figure 1B). Thus, MSFsc is a good marker for examining chronotypes. Participants with Japanese-style breakfast showed an earlier chronotype in both males and females (Figure 1C); on the other hand, the cereal group exhibited a later chronotype. Among male participants, there were significant differences (*p* < 0.05) between the Japanese and Japanese–Western-style groups (Figure 1C). The eating period refers to the time taken to consume each breakfast. The cereal group took a short time to consume breakfast compared with other breakfast groups (Figure 1D). Next, we examined the relationship between breakfast type and BMI (Figure 1E). Male participants had higher BMI values than female participants (Figure 1E). The cereal group showed lower BMI values in both sexes compared with the other breakfast groups (Figure 1E). When comparing METs values among breakfast styles, there were no significant differences between the groups (Figure 1F).

To identify the most important interaction in ongoing research, we investigated the interaction between sleep parameters and breakfast meal styles. Multivariable linear regression analysis was applied for the interaction between sleep parameters and breakfast meal style with the following confounding factors: age, sex, BMI, and total METs (Table 3). Breakfast intake was negatively associated with wake-up time on weekdays and weekends and MSFsc, and the Japanese meal style showed the largest values of the standardized coefficient (β) compared with other breakfast styles. These results suggest that participants who consume Japanese meals for breakfast display morningness.

In the next experiment, we investigated the association between the intake volume of nutritional components at breakfast or daily intake and breakfast style using Spearman’s analysis (Supplementary Table S1). In general, macronutrients such as protein, fat, carbohydrate, and sodium at breakfast time were positively and strongly associated with consuming a Japanese breakfast, whereas macronutrients were negatively associated with consuming cereal breakfast. Among micronutrients, vitamin K was positively correlated with Japanese breakfast and negatively correlated with cereal breakfast (Supplementary Table S1). Regarding the daily intake of nutrients, the positive and negative associations between breakfast style and nutritional components became weak and/or insignificant.

Since Spearman’s analysis revealed the positive and negative associations between breakfast style and nutritional components, in the next step, multivariate linear regression analysis was applied for the association between nutritional components at breakfast (Table 4) or at daily intake (Table 5) and breakfast meal style under confounding factors (age, sex, METs, and BMI). The standardized coefficient values of macronutrients, sodium, vitamin K, and dietary fiber were higher in the Japanese breakfast style group than in the other breakfast styles (Table 4). For example, the standardized coefficient values were 0.668, 0.432, 0.532, and 0.327 for carbohydrate intake in Japanese, Japanese–Western, Western, and cereal breakfasts, respectively (Table 4). Multivariable linear regression analysis was then applied to the level of daily intake of nutrients (Table 5). The standardized coefficient values of macronutrients, sodium, vitamin K, and dietary fibers were small and similar among all breakfast-style groups (Table 5). For instance, the standardized coefficient values were 0.201, 0.117, 0.196, and 0.031 for carbohydrate intake in the Japanese, Japanese–Western, Western, and cereal breakfast groups, respectively (Table 5).

**Figure 2:**
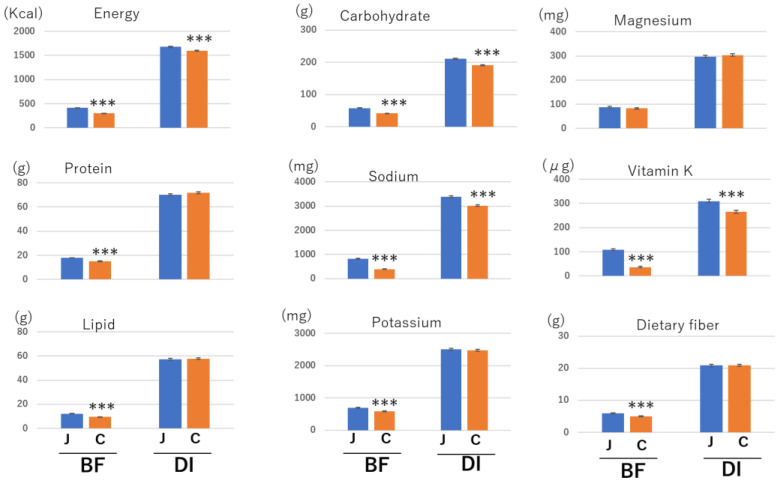
Intake of each nutrient in Japanese or cereal breakfast styles at breakfast and daily intake. All data were obtained from women. The columns represent the mean and standard errors. Two-group differences were statistically analyzed using the Student’s *t*-test. *** *p* < 0.001. J—Japanese meal; C—cereal meal. BF—breakfast; DI—daily intake.

In Table 4 and Table 5, it can be seen that the Japanese breakfast group may take more nutrients than the other breakfast-style groups on account of higher standardized coefficient values; therefore, we examined nutrition volumes at breakfast and daily intake. Since intake volumes were lower in the female than in the male group and the participant ratio of females was 70%, we applied this analysis only to females. In general, younger people consumed more food than older people, and the age of the Japanese breakfast group was similar to that of the cereal group, but not to the age of the Japanese–Western or Western groups (Figure 1B). Therefore, in this experiment, we compared the intake volume of nutrients between the Japanese breakfast and cereal breakfast groups of female participants. The intake volume of almost all nutrients except magnesium and vitamin E was higher in the Japanese breakfast group than in the cereal group at breakfast (Figure 2). At daily intake levels, intake volumes of carbohydrates, sodium, and vitamin K were still significantly higher in the Japanese breakfast group than in the cereal group (Figure 2). 

Each nutrient volume was an objective variable, and breakfast styles were explanatory variables. When multivariate linear regression analysis was applied, the four breakfast styles were analyzed simultaneously. For each subjective variable, the standardized coefficient (b) was indicated by a *p*-value. The R-squared and F-values were used to assess the fitness of the model. Confounding factors were age, sex, BMI, and total METs. Blue and pink columns indicate significant negative and positive interactions, respectively. J-W, Japanese and Western meals.

## 4. Discussion

To the best of our knowledge, this is the first cross-sectional study to evaluate the association of breakfast type and macronutrient intake with sleep parameters and the circadian clock, and no earlier study has investigated this important association in the past. Our results revealed that breakfast meal styles were well-associated with age, sex, BMI, sleep parameters, and the circadian clock. A study by Paoli Antonio et al. showed that the risk of weight gain was lower for those who consistently eat breakfast than for those who skip breakfast [25]. In addition, the results confirmed the link between meal timing and body weight, as well as the positive effects of consuming more calories in the morning [26]. Herein, by dividing breakfast meals into four categories, namely, Japanese, Japanese–Western (J/W), Western, and cereal meals, we showed that the association between macronutrient intake and breakfast style was higher in Japanese-style breakfast than in other types of breakfast. In addition, a higher volume of breakfast energy, protein, fat, carbohydrate, sodium, potassium, and vitamin K was observed in Japanese-style breakfast than in cereal breakfast. This may be related to the selection of various kinds of healthy food in Japanese breakfast, such as natto, rice, fish, and vegetables. Previously, a study that revealed the “Umami” definition in Japanese traditional meals has linked this finding with our study results [27]. One study showed that Japanese-style breakfast, which involves a large variety of foods and small portions, including miso soups and vegetables, ensures an adequate signal for satiety that prevents overeating, as well as a pleasing experience of consuming large amounts of bioactive compounds from vegetables [27]. Another study showed that Japanese-style meals include fish that contain high-quality proteins, eicosapentaenoic acid (EPA), docosahexaenoic acid (DHA), and omega-3 fatty acids [28]. Additionally, other results demonstrated that miso and tofu, which are soybean-based foods included in Japanese breakfast, can also reduce blood glucose and blood pressure [29,30]. In 2007, Shimazu et al. counted the Japanese-style diet as a low-fat diet that includes a high intake of fish and soybeans [31]. In this study, Japanese male participants were more likely to be Japanese breakfast eaters, since they showed higher BMI values than female participants. This issue highlights the importance of Japanese men being careful of their BMI. 

In a human experiment, the feeding pattern of three meals was delayed by 5 h under the same light–dark condition, and the clock gene expression pattern in peripheral tissues was examined accordingly [32]. A phase delay of 1–1.5 h was observed in the peripheral clock, suggesting that the phase of clock gene expression in peripheral tissue was determined by the timing of breakfast. Japanese breakfast eaters showed an earlier chronotype in both men and women, which is in line with other studies [33,34]. In the current study, the Japanese-style breakfast group showed earlier midpoints of sleep (MSFsc), suggesting that people preferring Japanese-type breakfast had morning chronotypes rather than evening chronotypes. Previous studies have only indicated a relationship between meal timing and the circadian clock [32,35]. Some studies have shown that participants who skip breakfast have a later chronotype [36,37]. Additionally, Toktas et al. showed that individuals with morning chronotypes consumed significantly more protein [38], which can also be seen in our study. In addition, Maukonen et al. found a significant association between fish intake and chronotypes. They stated that women with evening chronotypes had lower fish intake [39]. Owing to the cross-sectional design of the present study, the current results could not explain the cause and result of the interaction between Japanese breakfast and morning chronotypes. However, we could explain the possibility that people with a Japanese breakfast style exhibited a morning chronotype by the intake of each nutrient. In rodent experiments, carbohydrate- and protein-rich foods provided phase resetting by insulin [40] and IGF-1 [41]. DHA/EPA and fish oil also induced a phase reset of the mouse liver through the activation of insulin release [42]. Water-soluble fibers can reset the peripheral clock of the mouse liver through the production of short-chain fatty acids [43]. Vitamin K is a good treatment for patients with diabetes because of the release of insulin [44]. A high-salt diet ad libitum for over 2 weeks advanced the phase of clock gene expression by approximately 3 h in the liver, kidney, and lung [45]. In our current experiment, people with a Japanese breakfast style showed a high intake of carbohydrates, protein, salt, vitamin K (through natto production), and dietary fibers. Such characteristic breakfast eating patterns may be helpful for morningness in the circadian clock. 

When preparing a Japanese-type breakfast, adequate time is required for cooking and serving when compared with other breakfast types; for example, many bowls and plates are necessary to serve Japanese food compared with one plate for Western-type breakfast and one bowl for breakfast cereals. Thus, it is reasonable to wake up earlier to serve Japanese breakfast. Recently, a survey of breakfast, lunch, and dinner feeding habits was conducted among Japanese people. In that study, the time spent consuming breakfast, lunch, and dinner was 19, 25, and 34 min/d, respectively [46]. Thus, people who do not have enough time to serve meals, eat, and wash dishes in the morning may prefer Western-style breakfast, consume cereals and supplements, or skip breakfast. In the current study, we checked the time required to eat breakfast. The cereal breakfast-style group exhibited significantly quicker breakfast consumption than the other breakfast-style groups. A survey of breakfast was conducted among Japanese people, and the results demonstrated that feeding time clocks were longer—ranging from 5:00 to 10:00 (mean time, 7:24)—than lunchtime (range, 11:00–15:00, mean time, 12:29) [46], suggesting that people preferring breakfast at a later time may select Western and cereal breakfast types.

In the present experiments, age range analysis revealed that the Western-style breakfast group contained a higher number of elderly people than the Japanese-style group, and the cereal group contained a higher number of younger people. In agreement with the findings of a previous study [47,48], older people in our study had a morning preference. Therefore, we cannot explain why the morning preference observed in the Japanese-style breakfast group is directly associated with aging. However, the reason why people who prefer Western-style breakfast are older remains unknown. It could be because aging people avoid the complex service of Japanese-style breakfast by simply serving Western-style breakfast. 

The limitations of this study include misclassification due to self-reports, as well as unmeasured and uncontrolled confounders. Since this study had a cross-sectional design, we could not identify whether reverse causation had occurred therein. Further studies are required to understand the detailed mechanism of the relationships between breakfast type and macro- and micronutrient intake, with the circadian clock. Using data from an online survey in Japan, we identified that the midpoint of sleep, which indicates the human chronotype, was significantly associated with the type of breakfast intake in Japanese adults. This finding may contribute to the consideration of the relationship between chronotype and Japanese breakfast macronutrient intake, and it may reveal the characteristics of the chronotype. Japanese dietary habits are healthier and more different from those followed in other countries in terms of their long life expectancy and prevalence of diseases [27,49,50]. In summary, Japanese-style breakfast is recommended not only for healthy food but also for morning preference and a high intake of macro- and micronutrients.

## Figures and Tables

**Table 1:** Basic characteristics of participants.

	Male (Mean ± SD)	Female (Mean ± SD)	*p*
Number	766	1805	
Age	50.05 ± 10.9	44.3 ± 11.28	<0.0001
BMI	24.27 ± 3.3	22.78 ±3.8	<0.0001
Physical activity (METs)	38.04 ± 44.2	29.7 ± 39.3	<0.0001
BF style; Japanese (%)	223(29)	417(23)	0.001
BF style; J-W	69(9)	236(13)	0.003
BF style; Western	204(27)	543(30)	NS
BF style; Cereal	228(30)	558(31)	NS
weekday sleep onset ^(1)^	23.24 ± 0.84	23.81 ± 1.24	<0.0001
weekday wake-up ^(1)^	6.28 ± 1.26	6.67 ± 1.37	NS
free day sleep onset ^(1)^	23.75 ± 1.36	24.11 ± 1.32	<0.0001
free day wake-up ^(1)^	7.24 ± 1.45	7.80 ± 1.51	<0.0001
weekday sleep duration ^(2)^	6.73 ± 1.1	6.85 ± 1.12	0.0175
free day sleep duration ^(2)^	7.49 ± 1.24	7.68 ± 1.16	0.0001
MSFsc ^(1)^	3.06 ± 1.25	3.49 ± 1.26	<0.0001

^(1)^ clock time, ^(2)^ h.

**Table 2:** Spearman’s correlation analysis between basic characteristics, breakfast meal style, and sleep parameters.

	Sex	Age	BMI	Mets	Japanese	J-W	Western	Cereal
weekday sleep onset	−0.035	0.092 **	−0.034	0.061 **	0.020	0.008	0.013	−0.009
weekday wake-up	0.136 **	−0.222 **	0.071 **	−0.086 **	−0.090 **	0.001	−0.048 *	0.054 **
free day sleep onset	−0.050 *	0.070 **	−0.023	0.043 *	0.011	0.008	0.015	−0.014
free day wake-up	0.168 **	−0.295 **	0.071 **	−0.126 **	−0.093 **	−0.031	−0.049 *	0.079 **
weekday sleep duration	0.047 *	−0.109 **	0.013	−0.042 *	0.001	0.021	−0.032	−0.002
free day duration	0.075 **	−0.176 **	0.034	−0.099 **	0.000	−0.028	−0.032	0.019
MSFsc	0.161 **	−0.246 **	0.071 **	−0.092 **	−0.110 **	−0.010	−0.035	0.067 **

A higher score indicated a high association between the factors. Asterisks (* and **) in each column indicate the significance of the correlation (*p* < 0.05, *p* < 0.01). BMI, body mass index. METs and exercise parameters. MSFsc, a marker of morningness and evenness. J-W, Japanese and Western meals.

**Table 3:** Multivariate linear regression analysis for interaction between sleep parameters and breakfast meal style.

	Japanese		J-W		Western		Cereal			
	β	P	β	P	β	P	β	P	R2	F
weekday sleep onset	0.196	0.000	0.13	0.000	0.155	0.000	0.135	0.001	0.044	14.4
weekday wake-up	−0.327	0.000	−0.208	0.000	−0.289	0.000	−0.255	0.000	0.108	61.4
free day sleep onset	0.180	0.000	0.115	0.000	0.141	0.000	0.112	0.004	0.048	15.8
free daywake-up	−0.313	0.000	−0.212	0.000	−0.259	0.000	−0.219	0.000	0.155	57.0
weekday sleep duration	−0.074	0.053	−0.039	0.217	−0.092	0.021	−0.091	0.023	0.017	5.49
free day sleep duration	−0.064	0.088	−0.063	0.044	−0.079	0.044	−0.069	0.081	0.032	10.19
MSFsc	−0.334	0.000	−0.203	0.000	−0.264	0.000	−0.226	0.000	0.131	66.7

Each sleep parameter is an objective variable, and breakfast styles are explanatory variables. When multivariate linear regression analysis was applied, the four breakfast styles were analyzed simultaneously. For each subjective variable, the standardized coefficient (β) was indicated by a *p*-value. The R-squared and F-values were used to assess the fitness of the model. Confounding factors are age, sex, BMI, and total METs. J-W, Japanese and Western meals.

**Table 4:** Multivariate linear regression analysis for interaction between breakfast meal style and nutrients at breakfast.

Breakfast	Japanese		J-W		Western		Cereal			
	β	P	β	P	β	P	β	P	R2	F
Energy	0.647	0.000	0.429	0.000	0.548	0.000	0.316	0.000	0.239	96.5
protein	0.522	0.000	0.326	0.000	0.385	0.000	0.390	0.000	0.126	44.0
lipid	0.376	0.000	0.301	0.000	0.457	0.000	0.171	0.000	0.146	52.6
carbohydrate	0.668	0.000	0.432	0.000	0.532	0.000	0.327	0.000	0.225	89.1
sodium	0.566	0.000	0.340	0.000	0.282	0.000	0.057	0.107	0.278	118.1
potassium	0.502	0.000	0.309	0.000	0.331	0.000	0.387	0.000	0.122	42.3
calcium	0.241	0.000	0.170	0.000	0.268	0.000	0.352	0.000	0.060	19.7
magnesium	0.358	0.000	0.182	0.000	0.157	0.000	0.347	0.000	0.082	27.6
phosphorus	0.540	0.000	0.321	0.000	0.378	0.000	0.355	0.000	0.117	41.3
iron	0.208	0.000	0.121	0.000	0.118	0.004	0.278	0.000	0.031	9.90
zinc	0.225	0.000	0.125	0.000	0.096	0.019	0.181	0.000	0.043	13.1
vitamin A	0.170	0.000	0.129	0.000	0.120	0.003	0.194	0.000	0.027	8.75
vitamin D	0.018	0.657	−0.003	0.921	−0.028	0.499	0.046	0.270	0.008	2.46
vitamin E	0.048	0.231	0.048	0.139	0.032	0.440	0.081	0.052	0.006	2.02
vitamin K	0.501	0.000	0.206	0.000	0.064	0.083	0.063	0.090	0.202	78.5
vitamin B1	−0.039	0.324	−0.026	0.417	−0.034	0.410	0.012	0.778	0.008	2.40
vitamin B2	−0.042	0.291	−0.028	0.398	−0.059	0.153	0.007	0.867	0.005	1.69
niacin	−0.082	0.039	−0.066	0.042	−0.127	0.002	−0.082	0.048	0.008	2.35
vitamin B6	−0.025	0.528	−0.023	0.481	−0.034	0.412	0.019	0.650	0.005	1.60
vitamin B12	0.010	0.796	−0.011	0.739	0.005	0.913	0.007	0.874	0.005	1.67
folate	0.216	0.000	0.134	0.000	0.125	0.002	0.206	0.000	0.028	8.98
pantothenic acid	0.071	0.075	0.032	0.327	0.016	0.693	0.073	0.077	0.010	3.05
vitamin C	−0.012	0.754	−0.010	0.759	−0.049	0.233	0.058	0.159	0.013	4.17
dietary fiber	0.481	0.000	0.285	0.000	0.317	0.000	0.384	0.000	0.091	31.01

Each nutrient volume was an objective variable, and breakfast styles were explanatory variables. When multivariate linear regression analysis was applied, the four breakfast styles were analyzed simultaneously. For each subjective variable, the standardized coefficient (β) was indicated by a *p*-value. The R-squared and F-values were used to assess the fitness of the model. Confounding factors were age, sex, BMI, and total METs. J-W, Japanese and Western meals.

**Table 5:** Multivariate linear regression analysis for interaction between breakfast meal style and nutrients at daily intake.

Daily intake	Japanese		J-W		Western		Cereal			
	β	P	β	P	β	P	β	P	R2	F
Energy	0.168	0.000	0.084	0.002	0.164	0.000	0.054	0.123	0.249	103.3
protein	0.189	0.000	0.094	0.001	0.117	0.001	0.229	0.000	0.185	70.9
lipid	−0.010	0.769	−0.002	0.953	0.083	0.024	−0.018	0.634	0.163	60.9
carbohydrate	0.201	0.000	0.117	0.000	0.196	0.000	0.031	0.401	0.182	69.2
sodium	0.194	0.000	0.106	0.000	0.147	0.000	0.030	0.404	0.202	80.3
potassium	0.280	0.000	0.149	0.000	0.149	0.000	0.255	0.000	0.073	24.6
calcium	0.118	0.002	0.040	0.198	0.084	0.032	0.234	0.000	0.052	17.0
magnesium	0.152	0.000	0.008	0.800	−0.013	0.732	0.200	0.000	0.088	30.0
phosphorus	0.168	0.000	0.036	0.215	0.053	0.150	0.143	0.000	0.158	58.5
iron	0.022	0.564	−0.031	0.312	−0.043	0.272	0.142	0.000	0.043	13.9
zinc	0.081	0.028	−0.001	0.971	−0.026	0.491	0.086	0.025	0.079	26.6
vitamin A	0.002	0.950	−0.024	0.439	−0.072	0.067	0.033	0.402	0.022	7.02
vitamin D	0.011	0.773	−0.031	0.329	-0.032	0.424	0.056	0.163	0.013	4.01
vitamin E	0.027	0.475	0.003	0.916	0.002	0.967	0.072	0.074	0.008	2.45
vitamin K	0.283	0.000	0.093	0.002	0.051	0.190	0.145	0.000	0.065	21.8
vitamin B1	−0.040	0.294	−0.043	0.167	−0.070	0.078	0.026	0.522	0.016	5.00
vitamin B2	−0.039	0.304	−0.049	0.123	−0.080	0.044	0.026	0.519	0.014	4.60
niacin	−0.151	0.000	−0.129	0.000	−0.192	0.000	−0.101	0.011	0.037	12.0
vitamin B6	−0.037	0.331	−0.036	0.250	−0.059	0.139	0.036	0.363	0.015	4.80
vitamin B12	−0.001	0.982	−0.009	0.769	−0.022	0.587	0.003	0.940	0.008	2.70
folate	0.071	0.060	0.001	0.964	−0.016	0.676	0.125	0.002	0.042	13.5
pantothenic acid	−0.002	0.961	−0.033	0.290	−0.051	0.196	0.039	0.330	0.031	10.1
vitamin C	−0.019	0.622	−0.036	0.246	−0.069	0.082	0.071	0.076	0.020	6.22
dietary fiber	0.167	0.000	0.046	0.135	0.068	0.081	0.185	0.000	0.065	21.7

Each nutrient volume was an objective variable, and breakfast styles were explanatory variables. When multivariate linear regression analysis was applied, the four breakfast styles were analyzed simultaneously. For each subjective variable, the standardized coefficient (β) was indicated by a *p*-value. The R-squared and F-values were used to assess the fitness of the model. Confounding factors were age, sex, BMI, and total METs. J-W, Japanese and Western meals.

## Data Availability

Data will be sent on request from the corresponding author. The data are not publicly available because of patent preparation.

## Supplementary Materials

The following supporting information can be downloaded at: https://www.mdpi.com/article/10.3390/nu14173496/s1, Table S1: Spearman’s correlation analysis between breakfast meal style and nutrient intake.
