# Supplementary material for: Association of Japanese Breakfast Intake with Macro- and Micronutrients and Morning Chronotype
Source: Nutrients. 2022 Aug 25;14(17):3496. doi: 10.3390/nu14173496 (PMC9458211; doi:10.3390/nu14173496)
Supplement: Supplementary file 1 [file nutrients-14-03496-s001.zip › nutrients-1807447-supplementary.pdf]

Table S1. Spearman's correlation analysis between breakfast meal style and nutrient intake.

	Breakfast					Daily intake			
	Japanese	J-W	Western	Cereal		Japanese	J-W	Western	Cereal
Energy	.263**	.097**	.079**	-.270**		.102**	-0.014	.038*	-.100**
protein	.210**	.046*	-.055**	-.062**		.080**	-.043*	-.104**	.096**
lipid	.108**	.087**	.175**	-.243**		-0.004	-0.024	.056**	-.043*
carbohydrate	.278**	.094**	.062**	-.258**		.121**	0.008	.077**	-.159**
sodium	.378**	.165**	0.018	-.395**		.135**	0.022	0.009	-.126**
potassium	.180**	0.038	-.041*	-0.024		.106**	-.047*	-.076**	.069**
calcium	-0.033	-0.026	.048*	.111**		-0.009	-.072**	-.052**	.151**
magnesium	.195**	0.009	-.127**	.055**		.094**	-.080**	-.145**	.155**
phosphorus	.221**	.049*	-0.012	-.107**		.110**	-.063**	-.090**	.061**
iron	.142**	0.013	-.154**	.112**		.040*	-.068**	-.142**	.184**
zinc	.281**	.075**	-.120**	-.088**		.114**	-.039*	-.123**	.058**
vitamin A	.120**	.054**	-.059**	0.005		.043*	-0.035	-.090**	.102**
vitamin D	.182**	.040*	-.126**	0.008		.061**	-.050*	-.113**	.119**
vitamin E	.093**	.047*	-0.037	0.002		0.013	-.048*	-.080**	.123**
vitamin K	.425**	.157**	-.156**	-.264**		.202**	0.004	-.143**	0.002
vitamin B1	.054**	0.002	-.094**	.126**		0.005	-.057**	-.123**	.175**
vitamin B2	.062**	-0.007	-.086**	.113**		0.011	-.060**	-.132**	.180**
niacin	.179**	0.036	-.099**	-0.023		.050*	-.061**	-.125**	.127**
vitamin B6	.066**	-0.018	-.142**	.171**		0.003	-.059**	-.142**	.195**
vitamin B12	.232**	.061**	-.177**	-0.023		.083**	-0.022	-.123**	.063**
folate	.140**	0.035	-.081**	0.022		.046*	-.042*	-.112**	.125**
pantothenic acid	.173**	0.026	-.094**	0.002		.051**	-.048*	-.114**	.114**
vitamin C	0.029	0.011	-.068**	.111**		-0.006	-.049*	-.084**	.155**
dietary fiber	.213**	.039*	-.067**	-0.029		.093**	-.054**	-.092**	.094**

Higher score means highly association between breakfast meal style and daily intake , and other factors. Asterisks (* and **) in each column indicate the significance of correlation ($p < 0.05$ and $p < 0.01$), respectively. J-W, Japanese and Western meal.
